# Development and validation of a nomogram to predict recurrence for clinical T1/2 clear cell renal cell carcinoma patients after nephrectomy

**DOI:** 10.1186/s12893-024-02487-z

**Published:** 2024-06-26

**Authors:** Keruo Wang, Baoyin Guo, Yuanjie Niu, Gang Li

**Affiliations:** 1 Department of Urology, Tianjin Institute of Urology, The Second Hospital of Tianjin Medical University Tianjin, Tianjin, 300211 China; 2 Department of Urology, Tianjin Baodi Hospital, Baodi Clinical College of Tianjin Medical University, Tianjin, 301800 China

**Keywords:** Clear cell renal cell carcinoma, Nomogram, Recurrence-free survival, Nephrectomy, Validation

## Abstract

**Objective:**

To develop and validate a nomogram for predicting recurrence-free survival (RFS) for clinical T1/2 (cT1/2) clear cell renal cell carcinoma (ccRCC) patients after nephrectomy.

**Methods:**

Clinicopathological and survival data from 1289 cT1/2 ccRCC patients treated at the Second Hospital of Tianjin Medical University between 2017 and 2020 were included. Cox regression analysis was used to identify independent risk factors in 902 and 387 ccRCC patients in the training and validation cohorts, respectively, and construct the nomogram. The performance of the nomogram was assessed through calibration plots, time-dependent receiver operating characteristic (ROC) curves, C-index (concordance-index), and decision curve analysis (DCA). Kaplan-Meier curves were used to evaluate the probability of RFS in patients with different recurrence risks.

**Results:**

Age, tumor size, surgical approach, Fuhrman grade, and pT3a upstage were identified as independent predictors of RFS. The area under the curve (AUC) for the 3-year and 5-year RFS ROC curves were 0.791 and 0.835 in the training cohort, and 0.860 and 0.880 in the validation cohort. The DCA and calibration plots demonstrated the optimal application and excellent accuracy of the nomogram for predicting 3-year and 5-year RFS. Kaplan-Meier curves revealed significant differences in RFS among the three risk groups in both the training and validation cohorts. Clinically, the developed nomogram provides a more precise tool for risk stratification, enabling tailored postoperative management and surveillance strategies, ultimately aiming to improve patient outcomes.

**Conclusions:**

We developed a nomogram for predicting RFS in cT1/2 ccRCC patients after nephrectomy with high accuracy. The clinical implementation of this nomogram can significantly enhance clinical decision-making, leading to improved patient outcomes and optimized resource utilization in the management of ccRCC.

## Introduction

Renal carcinoma ranks as the third most prevalent genitourinary tumor globally, following prostate cancer and bladder cancer in frequency [1]. Renal cell carcinoma (RCC) constitutes approximately 90% of all renal carcinoma cases, with ccRCC accounting for 70-80% of RCC cases [2]. Extensive biological and clinical investigations have established that distinct histological subtypes of RCC exhibit specific clinical characteristics, biological behaviors, and genetic profiles, thereby yielding diverse oncological outcomes [3]. Notably, ccRCC, the most common histological subtype, exhibits more aggressive behavior compared to other subtypes. Moreover, one-third of ccRCC patients present with regional or distant metastasis at initial diagnosis, and 30–40% of localized ccRCC patients who undergo nephrectomy experience disease relapses [4].

The American Joint Committee on Cancer (AJCC) TNM staging system stands as the widely accepted method for prognostic prediction in RCC patients. Within this system, cT1/2 RCC is defined as a renal tumor confined to the kidney without local invasion or distant metastasis [5]. According to the TNM staging system, tumor size remains the sole distinguishing factor for T1a-T2b RCC. However, this approach does not account for the diverse pathological characteristics among cT1/2 ccRCC patients, which may lead to different clinical outcomes. Factors such as age, performance status, and tumor nuclear grade also affect the prognosis of ccRCC patients, highlighting the need for a comprehensive and accurate tool to assess the individual risk of RFS for cT1/2 ccRCC patients [6–8]. This underscores the necessity for a more comprehensive and accurate predictive tool. The need for such a tool is further emphasized by the limitations of current treatment options. Since RCC is insensitive to chemotherapy and radiotherapy, guidelines recommend partial nephrectomy (PN) and radical nephrectomy (RN) as standard treatment options. However, these procedures carry a 10% recurrence rate [9].

The European Association of Urology (EAU) guideline has recommended several prognostic nomogram models, including the UISS system, Leibovich prognostic score, VENUSS model, etc., which are considered accurate and commonly employed for localized RCC patients [10, 11]. However, these models often include broad patient populations and do not account for the unique clinical characteristics of specific patients. Furthermore, the predictive accuracy of these models diminishes over time, particularly beyond the initial years post-surgery [10, 11]. Given these limitations, there is a clear need for a more precise and tailored predictive tool for cT1/2 ccRCC patients. Our study addresses this gap by developing and validating a postoperative nomogram specifically for predicting RFS in cT1/2 ccRCC patients. By integrating a comprehensive range of factors, our model aims to provide more accurate individual risk assessments, ultimately guiding better clinical decision-making and improving patient outcomes.

## Patients and methods

### Study population

**Fig. 1:**
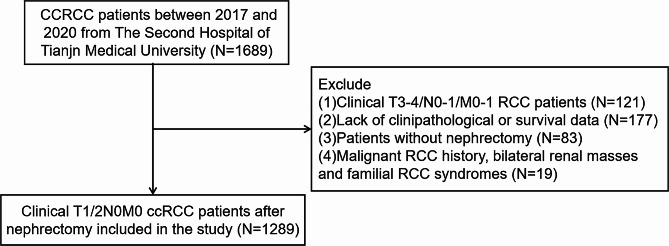
Flowchart of clinical T1/2 ccRCC patients after nephrectomy included in the study RCC, renal cell carcinoma; ccRCC, clear cell renal cell carcinoma

The study included all cT1/2N0M0 ccRCC patients who underwent surgical treatment (PN or RN) between January 2017 and December 2020 at The Second Hospital of Tianjin Medical University. The inclusion and exclusion criteria are shown in Fig. 1. In total, 1289 patients were included in the study and randomly divided into the training cohort (*n* = 902) and the validation cohort (*n* = 387) in a 7:3 ratio.

### Variables and outcomes

All related clinicopathological data of the patients were reviewed, and 18 variables were collected, including age, gender, laterality, body mass index (BMI), clinical symptoms (palpable mass, flank pain, gross hematuria), smoking history, hypertension, diabetes, hemoglobin levels, surgical approach, type of nephrectomy, RENAL score, TNM stage, tumor size, surgical margin status, Fuhrman grade, tumor necrosis and sarcomatoid differentiation. All patients received preoperative laboratory and imaging examinations. Histological subtypes were diagnosed according to the Heidelberg classification [12], and the nuclear grade was classified into low (grades I-II) and high (grades III-IV) groups based on the WHO Fuhrman nuclear grading system [13]. The imaging features of RCC patients were evaluated by two imaging specialists [14]. RFS was defined as the time from the date of renal tumor diagnosis to the date of RCC recurrence or the last follow-up. Censored data were referred to patients who did not experience relapse from the date of diagnosis to the date of last follow-up or December 1, 2022. Specifically, these patients were considered to have not experienced recurrence at the last point of contact. This approach ensures that the survival analysis accurately reflects the duration of RFS for all patients, including those without an event by the study’s end date.

### Statistical analysis

Statistical analysis was performed using SPSS 22.0 and R software (version 4.1.0). Categorical and continuous variables were described as frequencies (percentages) and medians (interquartile range [IQR]). The Chi-square test and Fisher’s exact test were used for analyzing categorical variables, while the t-test and Kruskal-Wallis test were used for comparing continuous variables. Univariate and multivariate Cox regression analyses were employed to determine the final factors for developing the nomogram to predict RFS probabilities.

The discriminatory ability of the nomogram was evaluated through internal and external validation using the C-index and time-dependent ROC curves. Calibration plots were used to assess the agreement between the predicted and actual probabilities of RFS. Furthermore, DCA was performed to evaluate the net benefit associated with using the nomogram. Based on the calculated risk score using the five independent predictors and related regression coefficients, patients were classified into low-risk, medium-risk, and high-risk groups. Kaplan-Meier curves and log-rank tests were used to assess the differences in survival outcomes. The rationale for selecting these specific methods was based on their suitability for the types of data and the research objectives.

## Results

### Baseline characteristics of the patients

**Fig. 2:**
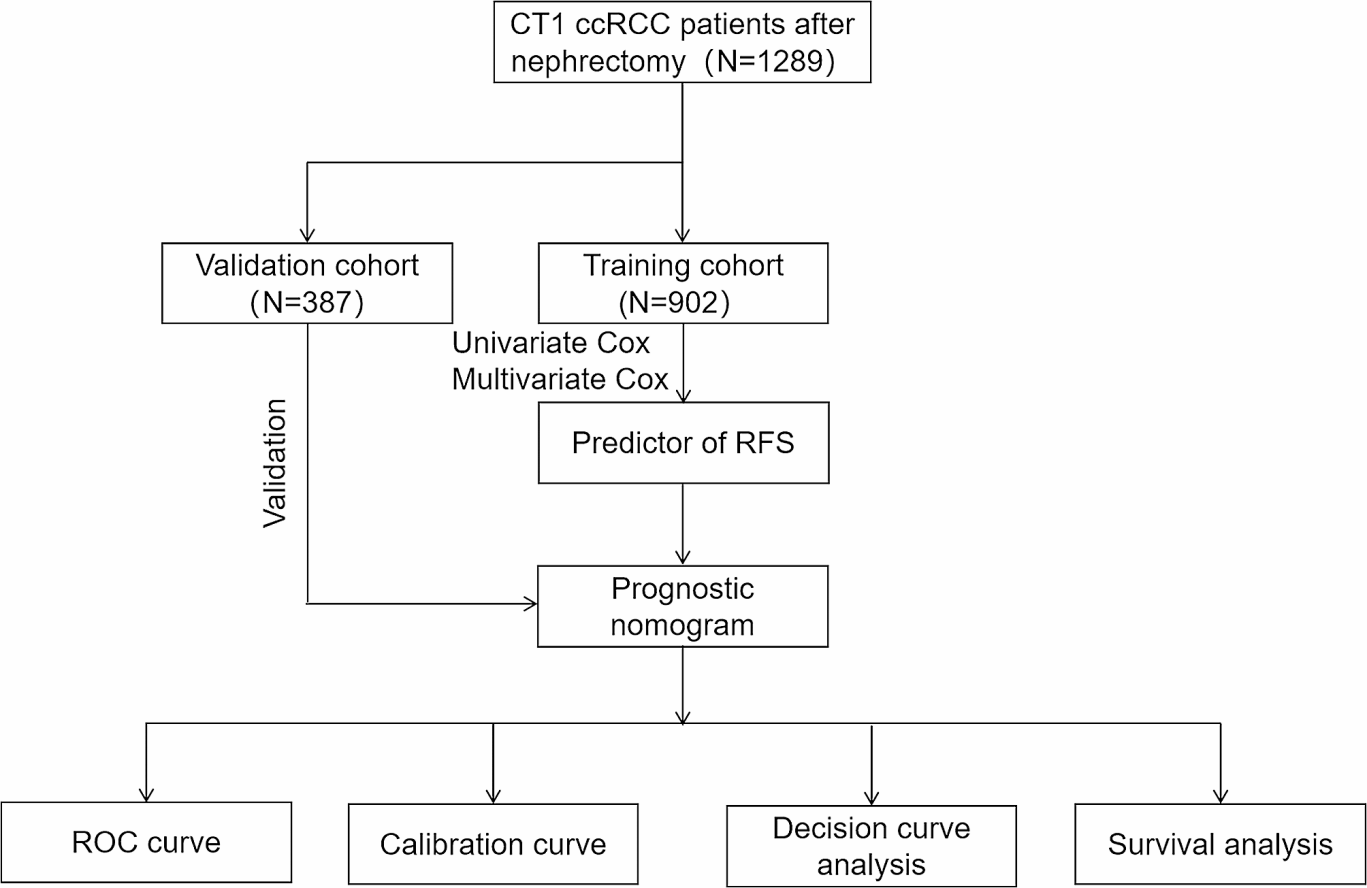
The workflow describing the overview of our study RCC, renal cell carcinoma; PN, partial nephrectomy; RN, radical nephrectomy; RFS, recurrence-free survival; ROC, receiver operating characteristic

The workflow of our study is shown in Fig. 2. A total of 1289 cT1/2 ccRCC patients who met the inclusion criteria were enrolled in the study and randomly assigned to a training cohort (*n* = 902) and a validation cohort (*n* = 387) at a 7:3 ratio. The baseline clinicopathologic and demographic characteristics of the 1289 eligible patients are reported in Table 1. There were no statistical differences in clinicopathological variables between training and validation groups. The median follow-up time was 33 (7–71) months for the training cohort and 34 (9–71) months for the validation cohort. The 1-year, 3-year, and 5-year RFS rates of patients were 98.6%, 92.7%, and 90.9% in the training cohort, while they were 99.0%, 92.3%, and 91.3% in the validation cohort.

**Table 1:** Clinicopathological features for cT1-2 ccRCC patients

Variables	Overall(*n* = 1289)	Training group(*n* = 902)	Validation group(*n* = 387)	*P*
**Preoperative parameters**				
Median age at surgery, year(IQR)	59.1(52.0–67.0)	59.0(52.0–67.0)	59.2(51.0–68.0)	0.765
Gender(%)				0.820
Male	905(70.2)	635(70.4)	270(69.8)	
Femal	384(29.8)	267(29.6)	117(30.2)	
BMI (kg/m^2^)(IQR)	25.8(23.4–27.7)	25.8(23.4–27.8)	25.7(23.5–27.7)	0.869
Laterality(%)				0.548
Left	636(49.3)	450(49.9)	186(48.1)	
Right	653(50.7)	452(50.1)	201(51.9)	
Smoking history(%)	491(38.1)	344(38.1)	147(38.0)	0.959
Clinical symptoms(%)				0.278
Palpable mass	4(0.4)	2(0.2)	2(0.5)	
Flank pain	287(22.3)	205(22.7)	82(21.2)	
Gross hematuria	146(11.3)	111(12.3)	35(9.0)	
Hypertension(%)	600(46.5)	409(45.3)	191(49.4)	0.186
Diabetes(%)	255(19.8)	175(19.4)	80(20.7)	0.600
Hemoglobin(g/dl)(IQR)	140.0(128.0-152.0)	139.9(127.0-151.3)	140.2(130.0-153.0)	0.888
cT stage (%)				0.330
cT1a	636(49.3)	435(48.2)	201(51.9)	
cT1b	485(37.6)	354(39.2)	131(33.9)	
cT2a	137(10.6)	92(10.2)	45(11.6)	
cT2b	31(2.5)	21(2.4)	10(2.6)	
RENAL score(%)				0.595
Low(4–6)	554(43.0)	382(42.4)	172(44.4)	
Moderate(7–9)	480(37.2)	344(38.1)	136(35.2)	
High(10–12)	255(19.8)	176(19.5)	79(20.4)	
Tumor size(cm)(IQR)	4.6(2.9–5.7)	4.6(2.9–5.6)	4.5(2.8–5.7)	0.521
**Surgical**				
Surgical approach(%)				0.171
Open	51(4.0)	30(3.3)	21(5.4)	
Laparoscopic	1113(86.3)	787(87.3)	326(84.3)	
Robotic	125(9.7)	85(9.4)	40(10.3)	
Type of nephrectomy(%)				0.620
Radical nephrectomy	503(39.0)	348(38.6)	155(40.1)	
Partial nephrectomy	786(61.0)	554(61.4)	232(59.9)	
**Pathologic**				
Surgical margin(%)				0.327
Positive	20(1.6)	12(1.3)	8(2.1)	
Negative	1269(98.4)	890(98.7)	379(97.9)	
Tumor grade(%)				0.640
Low(I-II)	985(76.4)	686(76.0)	299(77.3)	
High(III-IV)	304(23.6)	216(24.0)	88(22.7)	
Tumor necrosis(%)	195(15.1)	141(15.6)	54(14.0)	0.447
2010 pT stage				0.524
pT1a	625(48.5)	428(47.5)	197(50.9)	
pT1b	429(33.3)	314(34.8)	115(29.7)	
pT2a	100(7.8)	68(7.5)	32(8.3)	
pT2b	21(1.6)	14(1.6)	7(1.8)	
pT3	114(8.8)	78(8.6)	36(9.3)	
Sarcomatoid differentiation(%)				0.732
Yes	10(0.8)	8(0.9)	2(0.5)	
No	1279(99.2)	894(99.1)	385(99.5)	

ccRCC: clear cell renal cell carcinoma

### Identification of the significant predictors

**Fig. 3:**
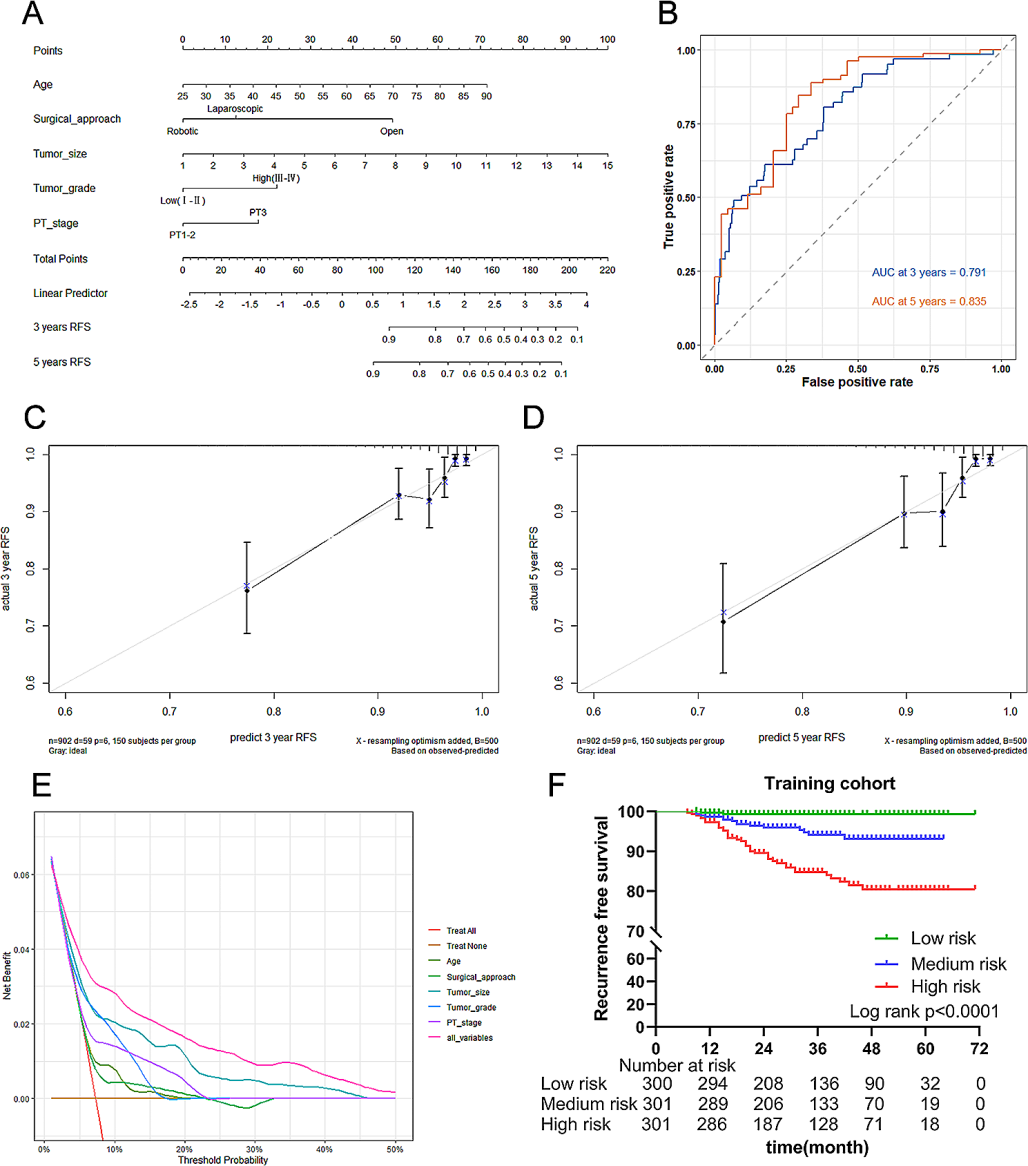
(**A**) Nomogram for 3-year and 5-year prediction of RFS, (**B**) time-dependent ROC curves of 3-year and 5-year RFS, (**C-D**) calibration plot of 3-year and 5-year RFS, (**E**) DCA, (**F**) Kaplan-Meier curves compare the difference of RFS among the low, medium and high-risk groups in the training cohort RFS, recurrence-free survival; ROC, receiver operating characteristic; AUC, area under the curve; DCA, decision curve analysis

We initially conducted univariate Cox analysis on the original 18 variables and excluded those with *P* > 0.1 (Table 2). Subsequently, multivariate Cox analysis was performed to screen the remaining 10 variables and identify the significant predictors of RFS in the training cohort. The result of the multivariate Cox analysis indicated that age (*P* = 0.008), tumor size (*P* < 0.001), surgical approach (*P* = 0.011), Fuhrman grade (*P* = 0.014), and pT3a upstage (*P* = 0.074) were independent predictors of RFS in cT1/2 ccRCC patients after nephrectomy in the training cohort (Table 2). Table 3 shows the results of the univariate and multivariate Cox regression analyses of RFS in the validation cohort. No statistically significant collinearity was observed among the predictors, and a significance level of *p* < 0.1 was considered statistically significant. A comparison of the two predictive models with multivariate Cox regression inclusion criteria of *p* < 0.1 and *p* < 0.05 in the training cohort revealed that the inclusion of pT3a upstage was the difference between the two models. The results showed that the 3-year and 5-year AUC of ROC curves were 0.791 and 0.835 in the nomogram model with pT3a upstage (Fig. 3B), and 0.785 and 0.826 in that without pT3a stage (Figure not shown), which showed better discrimination of the former nomogram. Additionally, the nomogram model with pT3a upstage exhibited better calibration (AIC, Akaike information criterion 705.66 vs. 706.58) than the model without pT3a stage in the training cohort.

**Table 2:** Univariate and multivariate Cox regression analyses of RFS for cT1-2 ccRCC patients in the training cohort

Variables	Univariate			Multivariate		
	HR	95%CI	*P* value	HR	95%CI	*P* value
**Preoperative parameters**						
Age at surgery	**1.039**	**1.013–1.066**	**0.003**	**1.036**	**1.009–1.063**	**0.008**
Gender			0.416			
Male	1.276	0.710–2.292				
Female	1(Reference)					
BMI	1.024	0.957–1.097	0.492			
Laterality			0.893			
Left	0.965	0.579–1.608				
Right	1(Reference)					
Smoking history	0.999	0.592–1.686	0.998			
Clinical symptoms	**2.322**	**1.389–3.884**	**0.001**			
Hypertension	1.287	0.772–2.144	0.333			
Diabetes	1.300	0.713–2.368	0.391			
Hemoglobin	**0.985**	**0.973–0.996**	**0.011**			
RENAL score			**< 0.001**			
Low(4–6)	**1(Reference)**					
Moderate(7–9)	**1.620**	**0.780–3.363**	**0.196**			
High(10–12)	**5.359**	**2.734–10.504**	**< 0.001**			
Tumor size	**1.350**	**1.247–1.461**	**< 0.001**	**1.252**	**1.143–1.372**	**< 0.001**
**Surgical**						
Surgical approach			**< 0.001**			**0.011**
Open	**1(Reference)**			**1(Reference)**		
Laparoscopic	**0.193**	**0.091–0.408**	**< 0.001**	**0.317**	**0.145–0.693**	**0.004**
Robotic	**0.092**	**0.024–0.349**	**< 0.001**	**0.213**	**0.054–0.843**	**0.028**
Type of nephrectomy			**< 0.001**			
Radical nephrectomy	**2.997**	**1.759–5.108**				
Partial nephrectomy	**1(Reference)**					
**Pathologic**						
Surgical margin			0.066			
Negative	1(Reference)					
Positive	3.768	0.918–15.465				
Fuhrman grade			**< 0.001**			**0.014**
I-II	**1(Reference)**			**1(Reference)**		
III-IV	**3.589**	**2.154–5.981**		**2.004**	**1.149–3.495**	
Tumor necrosis	**2.473**	**1.420–4.305**	**0.001**			
pT stage			**< 0.001**			**0.074**
pT1-2	**1(Reference)**			**1(Reference)**		
pT3	**4.052**	**2.254–7.283**		**1.761**	**0.947–3.273**	
Sarcomatoid differentiation	2.252	0.312–16.266	0.421			

ccRCC: clear cell renal cell carcinoma. *P* < 0.1 is considered as statistical significance

**Table 3:** Univariate and multivariate Cox regression analyses of RFS for cT1-2 ccRCC patients in the validation cohort

Variables	Univariate			Multivariate		
	HR	95%CI	*P* value	HR	95%CI	*P* value
**Preoperative parameters**						
Age at surgery	**1.070**	**1.029–1.113**	**0.001**	**1.047**	**1.002–1.095**	**0.041**
Gender			0.239			
Male	1.797	0.678–4.765				
Female	1(Reference)					
BMI	0.943	0.843–1.055	0.309			
Laterality			0.457			
Left	0.744	0.342–1.620				
Right	1(Reference)					
Smoking history	1.224	0.562–2.665	0.611			
Clinical symptoms	0.765	0.322–1.821	0.545			
Hypertension	0.710	0.326–1.547	0.389			
Diabetes	1.301	0.522–3.241	0.573			
Hemoglobin	**0.960**	**0.942–0.979**	**< 0.001**			
RENAL score			**< 0.001**			
Low(4–6)	**1(Reference)**					
Moderate(7–9)	**1.609**	**0.491–5.272**	**0.433**			
High(10–12)	**8.393**	**3.043–23.150**	**< 0.001**			
Tumor size	**1.403**	**1.254–1.570**	**< 0.001**	**1.269**	**1.105–1.458**	**0.001**
**Surgical**						
Surgical approach			**0.001**			**0.016**
Open	**1(Reference)**			**1(Reference)**		
Laparoscopic	**0.168**	**0.066–0.430**	**< 0.001**	**0.275**	**0.104–0.727**	**0.009**
Robotic	**0.337**	**0.095–1.196**	**0.093**	**0.781**	**0.205–2.968**	**0.717**
Type of nephrectomy			**0.005**			
Radical nephrectomy	**3.164**	**1.410–7.100**				
Partial nephrectomy	**1(Reference)**					
**Pathologic**						
Surgical margin			0.421			
Negative	1(Reference)					
Positive	2.276	0.307–16.843				
Fuhrman grade			**< 0.001**			**< 0.001**
I-II	**1(Reference)**			**1(Reference)**		
III-IV	**5.993**	**2.720-13.207**		**5.290**	**2.106–13.286**	
Tumor necrosis	**2.279**	**0.958–5.422**	**0.063**			
pT stage			**< 0.001**			**0.094**
pT1-2	**1(Reference)**			**1(Reference)**		
pT3	**5.993**	**2.720-13.207**		**2.039**	**0.886–4.691**	
Sarcomatoid differentiation	**8.325**	**1.126–61.529**	**0.038**			

ccRCC: clear cell renal cell carcinoma. *P* < 0.1 is considered as statistical significance

### Nomogram development and validation

We constructed a prognostic nomogram for 3-year and 5-year RFS probabilities (Fig. 3A), which consisted of one demographic variable (age) and four clinicopathological variables (tumor size, pT3a upstage, surgical approach, and tumor grade). Each variable corresponded to a point on the “Points” scale based on its contribution to the survival outcome, and the total points were obtained by summing the points of all variables. Then, the probability of individual 3-year and 5-year RFS can be calculated by drawing down a vertical line from the location of the nomogram on the horizontal axis labeled “points”.

**Fig. 4:**
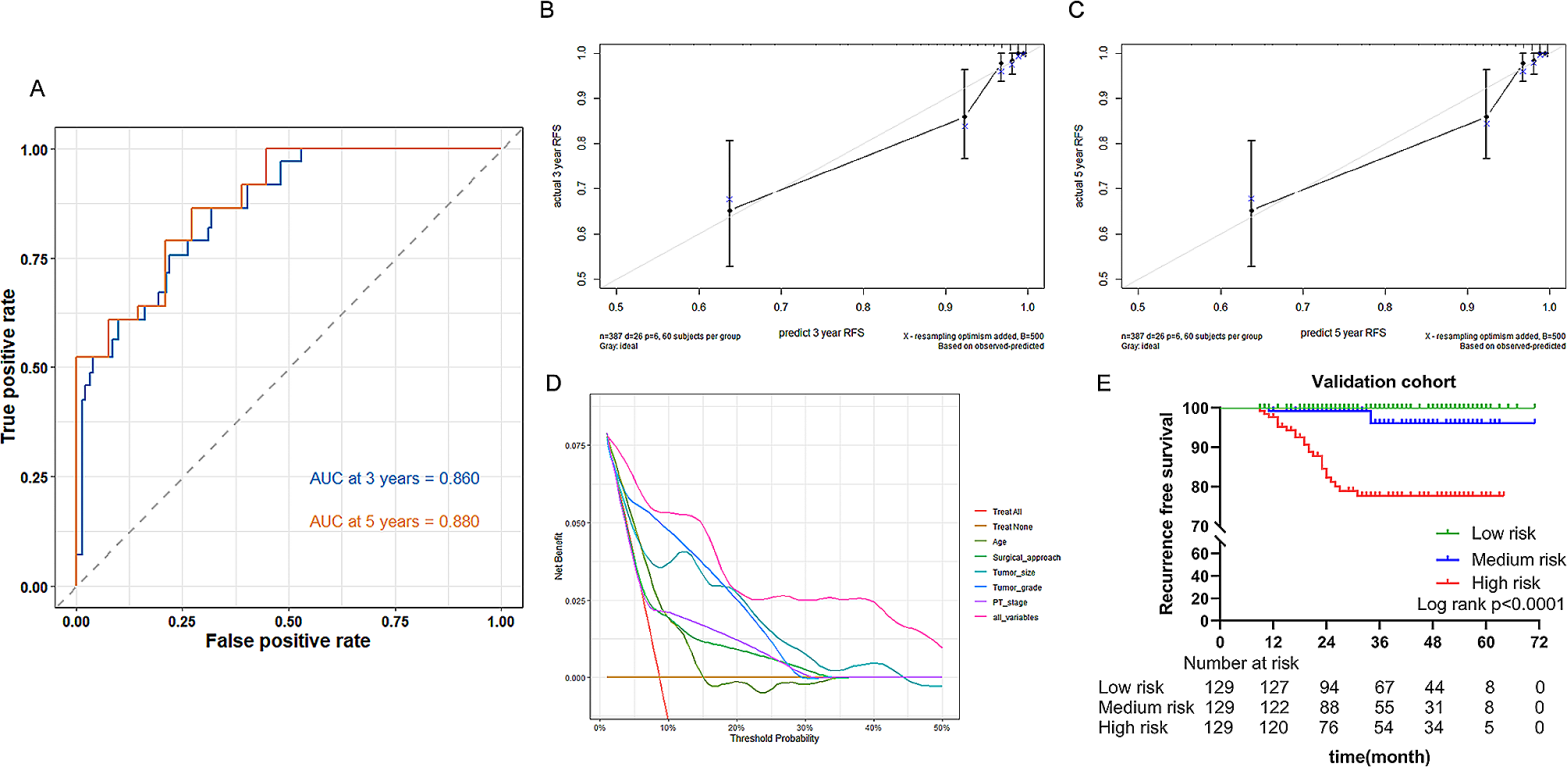
(**A**) time-dependent ROC curves of 3-year and 5-year RFS, (**B-C**) calibration plot of 3-year and 5-year RFS, (**D**) DCA, (**E**) Kaplan-Meier curves compare the difference of RFS among the low, medium and high-risk groups in the validation cohort RFS, recurrence-free survival; ROC, receiver operating characteristic; AUC, area under the curve; DCA, decision curve analysis

The C-index of the established nomogram was 0.797 in the training cohort and 0.871 in the validation cohort. As depicted in Figs. 3B and 4A, the AUC of 3-year and 5-year ROC curves showed the excellent discrimination ability of the nomogram in both the training cohort (3-year: 0.791, 5-year: 0.835) and the validation cohort (3-year: 0.860, 5-year: 0.880). Furthermore, calibration curves for 3-year and 5-year RFS illustrated considerable agreement between the actual and predicted probabilities, indicating good calibration of the nomogram in both the training and validation cohorts (Figs. 3C-D and 4B-C). As shown in Figs. 3E and 4D, the results of DCA showed that the nomogram had a positive net benefit in predicting RFS, highlighting its superior clinical application compared to the TNM staging system in both cohorts.

### Risk stratification model

According to the individual risk scores calculated by the novel nomogram, we built a risk stratification model. CT1/2 ccRCC patients were divided into low, medium, and high-risk groups on average. The result of Kaplan-Meier curves and log-rank tests showed that the differences in RFS were statistically significant both in the training (*P* < 0.0001) and validation (*P* < 0.0001) cohorts (Figs. 3F and 4E). The 3-year RFS probabilities of low, medium, and high-risk groups were 99.3%, 94.1%, and 84.8% in the training cohort, and the 5-year RFS probabilities were 99.3%, 93.1%, and 80.5%.

## Discussion

As the most common subtype of RCC, ccRCC is also known for its aggressive behavior. Although compliance with the postoperative follow-up guidelines, about one-third of ccRCC patients experience disease recurrence. As a consequence, precise prediction of the individual risk of renal tumor recurrence after nephrectomy is important to develop treatment plans and make individual surveillance [15]. Age, tumor size, sarcomatoid features, tumor nuclear grade, tumor necrosis, surgical margin status, and some other factors have been reported to be potentially related to RFS for localized ccRCC patients [11, 16, 17]. However, there remains some controversy regarding the independent prognostic factors for cT1/2 ccRCC patients treated with nephrectomy. Our study identified age, tumor size, surgical approach, Fuhrman grade, and pT3a upstage as independent risk factors.

Age has consistently been recognized as an important prognostic factor in many different kinds of cancers, and is also regarded as a risk stratification factor for RCC patients [18]. Recently, Liao et al. evaluated risk factors for ccRCC patients across different age groups using the SEER database and identified age as an independent predictor of overall survival (OS) and cancer-specific survival (CSS) [19]. They revealed the optimal cut-off values for age were 58 and 76 years for OS, and 51 and 76 years for CSS. Moreover, Saeed et al. analyzed oncologic outcomes for localized ccRCC patients based on the RECUR database [20]. They observed that ccRCC patients above 75 years of age had a significantly higher risk of death from RCC recurrence compared to patients aged 18–60 and 60–75 years. Consistent with existing studies, our study defined age as a continuous variable and found it to be an independent predictor of RFS for cT1/2 ccRCC patients. Subsequently, our study also revealed that tumor size is a major predictor affecting disease recurrence for cT1/2 ccRCC patients after nephrectomy. The recurrence rate increased by approximately 30% for each 1 cm increase in tumor size, which is consistent with findings from numerous previous studies [21, 22]. Michael et al. reviewed 1809 pT1, pT2, and pT3a RCC patients, investigating the relationship between the risk of disease recurrence and tumor size, pT stage [23]. They found that increasing tumor size had a higher risk of recurrence, regardless of tumor stage (*p* < 0.0001). Several newly established nomogram models for predicting recurrence in RCC patients also include tumor size as a predictor [24, 25]. With the advancement of surgical techniques, laparoscopic and robotic nephrectomy have become widely used in the treatment of localized RCC patients. Previous observational studies have shown conflicting oncologic outcomes between minimally invasive and open surgery [26, 27]. In our study, cT1/2 ccRCC patients who underwent open nephrectomy generally had more complex renal masses compared to those who underwent laparoscopic or robotic nephrectomy, potentially leading to a higher risk of disease recurrence. Possible explanations for the RFS advantage of minimally invasive nephrectomy include decreased postoperative morbidity, declined surgical stress, and cumulative surgeon experience [26].

Among all the postoperative risk factors, Fuhrman grade and pT3a upstage were identified as key factors for constructing the nomogram. Fuhrman grade has been used as an independent predictor of disease recurrence for RCC patients and included in the predictive models [28]. As mentioned earlier, TNM staging has been externally validated for accurately stratifying the RFS of ccRCC patients, and patients with more advanced tumor or nodal stages are more likely to experience worse oncologic outcomes [16]. One previous study evaluated whether incidental pT3a upstaging for cT1 RCC after PN resulted in inferior oncologic outcomes compared to pT1a-b disease, and the result suggested that upstaging could increase the risk of local recurrence and lead to reduced survival [29]. Lee et al. indicated that patients with cT1 upstaging to pT3 had poorer RFS, CSS, and OS compared to non-upstaging patients [30]. In our study, we found that upstaging was an independent prognostic factor for RFS in cT1/2 ccRCC patients after nephrectomy.

To our acknowledgment, several predictive models have been established to predict survival outcomes for RCC patients, and two of them have been developed specifically for predicting RFS in localized ccRCC patients: the University of California, Los Angeles, Integrated Staging System (UISS) and the Stage, Size, Grade, and Necrosis (SSIGN) score [31]. Both models have been externally validated, with the former including ECOG PS, TNM stage, and tumor nuclear grade, and the latter including TNM stage, tumor size, tumor nuclear grade, and tumor necrosis [15, 31]. Previous studies have evaluated the discriminative ability of these models and found that their predictive ability is highest within the first 2 years and then decreases over time. The C-index of these models ranges from 0.556 to 0.760 [15, 31, 32]. The major advantages of our nomogram are as follows: Firstly, it is the first predictive model specially developed for cT1/2 ccRCC patients after nephrectomy, whose RFS rate is typically underestimated. Secondly, In addition to the pathological data (Fuhrman grade, pT stage, tumor necrosis, etc.), we also collected additional data potentially related to RFS, including imaging data such as the RENAL score, laboratory data such as hemoglobin levels, clinical symptoms, and chronic disease. These factors were not included in the other prediction models. Finally, the accuracy of our nomogram is significantly higher than that of the two existing models. Furthermore, the C-index, AUC of time-dependent ROC curves, calibration plots, and DCA curves all demonstrate the discrimination, predictive ability, and clinical application of our nomogram in various ways.

Although our study developed the first nomogram for predicting RFS in cT1/2 ccRCC patients after nephrectomy, some limitations should also be noted. Primarily, the nomogram for 1-year RFS was not developed due to the limited number of patients experiencing relapse within the 1-year follow-up period. Secondly, the patients enrolled in our retrospective study were from single-center, which may exist selection bias. Finally, the validation cohort in our study was on the basis of a small sample data and our nomogram requires further external or multi-center validation. In future research, we plan to include additional variables such as known tumor history and genetic syndromes [33]. We will also compare the effects of different gas insufflation systems and the clamp techniques on the prognosis of cT1/2 ccRCC patients [34, 35].

## Conclusion

Age, tumor size, pT3a upstage, Fuhrman grade, and surgical approach were found to be significantly associated with the RFS of cT1/2 ccRCC patients after nephrectomy. This study presents the first predictive nomogram for calculating the probability of 3-year and 5-year RFS in cT1/2 ccRCC patients, offering a more accurate and reliable tool for risk stratification and prognosis assessment compared to the traditional TNM staging system. The implementation of this predictive model can assist urologists in clinical decision-making and the development of personalized treatment plans, ultimately leading to improved patient outcomes.

## Data Availability

The datasets used and/or analyzed during the current study are available from the corresponding author on reasonable request.

## Abbreviations

RFS: Recurrence-free survival
CCRCC: Clear cell renal cell carcinoma
ROC: Receiver operating characteristic
C-index: Concordance-index
DCA: Decision curve analysis
AUC: Area under the curve
RCC: Renal cell carcinoma
PN: Partial nephrectomy
RN: Radical nephrectomy
AJCC: American Joint Committee on Cancer
EAU: European Association of Urology
BMI: Body mass index
IQR: Interquartile range
AIC: Akaike information criterion
OS: Overall survival
CSS: Cancer-specific survival
UISS: The University of California, Los Angeles, Integrated Staging System
SSIGN: Stage, Size, Grade, and Necrosis
